# Plasma sterol profiling in autism spectrum disorder: insights from cerebrotendinous xanthomatosis screening and beyond

**DOI:** 10.1007/s11011-026-01827-7

**Published:** 2026-03-18

**Authors:** Kagan Calisgan, Tanyel Zubarioglu, Esra İsat, Hanim Babazade, Sedanur Akca-Yesil, Selin Akbulut, Esma Uygur, Vildan Keskin, Busra Cirkin, Gizem Durcan, Burak Dogangun, Mehmet Serif Cansever, Ayse Cigdem Aktuglu-Zeybek, Ertugrul Kiykim

**Affiliations:** 1 Department of Pediatric Nutrition and Metabolism, İstanbul University-Cerrahpaşa, Cerrahpaşa Medical Faculty, İstanbul, 34098 Turkey; 2 Research Laboratory of Metabolism, İstanbul-University-Cerrahpaşa, Cerrahpaşa Medical Faculty, İstanbul, Turkey; 3 Nutrition and Dietetics Unit, İstanbul-University-Cerrahpaşa, Cerrahpaşa Medical Faculty, İstanbul, Turkey; 4 Department of Psychiatric Nursing, Institute of Health Sciences, Marmara University, İstanbul, Turkey; 5 Department of Child and Adolescent Psychiatry, Istanbul University-Cerrahpaşa, Cerrahpaşa Medical Faculty, İstanbul, Turkey; 6 Department of Medical Laboratory Techniques, The Vocational School of Health Services, Istanbul University-Cerrahpaşa, İstanbul, Turkey

**Keywords:** Cerebrotendinous xanthomatosis, Autism spectrum disorder, Phytosterol, Screening, Bile acid

## Abstract

Cerebrotendinous xanthomatosis (CTX) is a rare, treatable bile acid synthesis disorder characterized by increased levels of cholestanol. Studies indicate that autism spectrum disorder (ASD) may be an early manifestation of CTX. Independent of CTX, disturbances in sterol and bile acid metabolism are observed in ASD. Therefore, this study aimed to estimate the prevalence of CTX in a pediatric ASD cohort using cholestanol-based screening with reflex *CYP27A1* sequencing and to compare plasma sterol profiles among children with ASD. We conducted a single-center, cross-sectional study including 103 patients with ASD and 70 age-matched, normally developed children. Fasting plasma cholestanol, campesterol, stigmasterol and sitosterol were quantified by gas chromatography/mass spectrometry. Participants with cholestanol ≥ 7 µg/ml underwent *CYP27A1* sequencing, and five-day dietary recalls were analyzed in 75 ASD participants. Elevated cholestanol was observed in 27 of 103 patients with ASD (26.2%) but in none of the controls (*p* < 0.001). No participant had biallelic pathogenic *CYP27A1* variants; one heterozygous variant of uncertain significance was detected. Median concentrations of cholestanol, campesterol, sitosterol and stigmasterol were significantly higher in patients with ASD than in controls (all *p* ≤ 0.001), and sterol fractions were strongly correlated (cholestanol–campesterol *r* = 0.74, *p* < 0.001). In summary, no cases of CTX were identified in this cohort, suggesting that cholestanol-based screening in unselected ASD populations may have limited yield, particularly in the absence of additional clinical features suggestive of CTX. Nevertheless, the elevated plasma sterol levels in ASD patients suggest dysregulated sterol and bile acid homeostasis, warranting further investigation.

**Supplementary Information:**

The online version contains supplementary material available at 10.1007/s11011-026-01827-7.

## Introduction

Cerebrotendinous xanthomatosis (CTX) is a rare inherited metabolic disease caused by biallelic mutations in the *CYP27A1* gene, which encodes the mitochondrial enzyme sterol 27-hydroxylase (Federico and Gallus 1993). This enzyme is essential for the proper functioning of both the classical and alternative bile acid synthesis pathways. Impaired function of this enzyme reduces bile acid synthesis - particularly of chenodeoxycholic acid (CDCA) - and leads to an accumulation of toxic bile acid precursors such as cholestanol (Federico and Gallus 1993; Nie et al. 2014). Clinically, CTX is characterized by a combination of neurological and systemic features. Early signs include neonatal cholestasis, chronic diarrhea, and bilateral juvenile cataracts. As the disease progresses, patients may develop pyramidal and cerebellar signs, cognitive impairment, psychiatric symptoms and tendon xanthomas (Nie et al. 2014). Treatment consists of replacement of the end product CDCA, which normalizes the bile acid pool and can prevent or even reverse symptoms if introduced early despite being a treatable condition, CTX remains significantly underdiagnosed, with an average diagnostic delay more than 2 decades, often leading to irreversible neurological damage (Mignarri et al. 2014; Stelten et al. 2021; Zubarioglu et al. 2025).

Autism spectrum disorder (ASD) is a heterogeneous neurodevelopmental disorder characterized by persistent deficits in social communication and interaction as well as restrictive, repetitive patterns of behavior, interests or activities (Harm et al. 2013). The estimated prevalence is around 1 in 31 children, with a notable male predominance (Shaw 2025). Early diagnosis and timely implementation of evidence-based interventions are critical to improving adaptive functioning and long-term outcomes (Smith 1999; Camarata 2014). While ASD is widely recognized as a complex disorder with multifactorial etiology, converging evidence points to the interaction of polygenic susceptibility with various environmental factors, particularly during critical windows of neurodevelopment. In addition to monogenic causes and chromosomal syndromes, recent research has identified a number of biological systems - including immune system dysregulation, mitochondrial dysfunction, oxidative stress, and gut–brain axis dysfunction - as potential factors in the pathophysiology of ASD (Park et al. 2016; Xu et al. 2019; Oge-Enver et al. 2023; Ayoub 2025). From an etiological perspective, inherited metabolic diseases (IMDs), although individually rare, represent a clinically relevant subgroup, as certain IMDs can either mimic ASD phenotypes or exacerbate behavioral and cognitive symptoms (Kiykim et al. 2016; Senarathne et al. 2023).

Cerebrotendinous xanthomatosis is one of the IMDs that have been reported in association with ASD, especially in early stages of the disease (Stelten et al. 2018). A small number of case reports and observational studies have indicated that ASD-like features may occasionally be the first neurological manifestation of CTX. Of note, these behavioral manifestations may show improvement with early initiation of CDCA therapy, highlighting the potential reversibility of some neuropsychiatric symptoms. However, CTX is not currently included in routine metabolic screening protocols for children with ASD and few studies have investigated this potential overlap (Karadag et al. 2024).

Given the treatable nature of CTX and the reported association with ASD, the primary aim of this study was to screen for potential CTX cases in a pediatric ASD cohort by measuring plasma cholestanol and associated phytosterol levels, followed by *CYP27A1* gene analysis if indicated. In addition, we aimed to determine whether sterol metabolism is altered in children with ASD by examining plasma concentrations of phytosterols, with the broader goal of understanding potential metabolic contributions to the pathophysiology of ASD.

## Materials and methods

### Study design and participants

This single-center cross-sectional study was conducted between January 2023 and January 2025 at Istanbul University-Cerrahpaşa, Cerrahpaşa Faculty of Medicine, Departments of Pediatric Nutrition and Metabolism. The study included a patient group with a confirmed diagnosis of ASD and a control group of typically developed children.

The inclusion criteria for the ASD patients were being under 18 years of age and had a confirmed diagnosis of ASD according to the DSM-5 diagnostic criteria provided by a child psychiatrist. The control group consisted of age-matched typically developed children with no history of chronic disease.

The exclusion criteria for the participants were as follows:

- Having an underlying inherited metabolic or genetic disorder that may have symptoms associated with ASD or contribute to its phenotypic presentation.
- Severe visual and hearing impairments.
- Taking medications known to affect plasma cholestanol levels, such as corticosteroids or ezetimibe.
- Secondary conditions known to affect cholestanol levels, e.g. liver disease, familial hypercholesterolemia, sitosterolemia or cholestasis.

The primary objective of this study was to estimate the prevalence of CTX in patients diagnosed with ASD. Using the standard formula for single-proportion estimation with a 95% confidence level (type I error of 0.05) and a margin of error of ± 3%, the minimum sample size required was determined to be 84 patients. An additional loss of 20% of cases was also included in the sample size so that the final sample size was 103 cases.

Ethical approval ffor the study was obtained from the local ethics committee (approval number: E-22686390-050.01.04-11880). Informed consent was obtained from the parents of all participants.

### Details of the study period

**Fig. 1:**
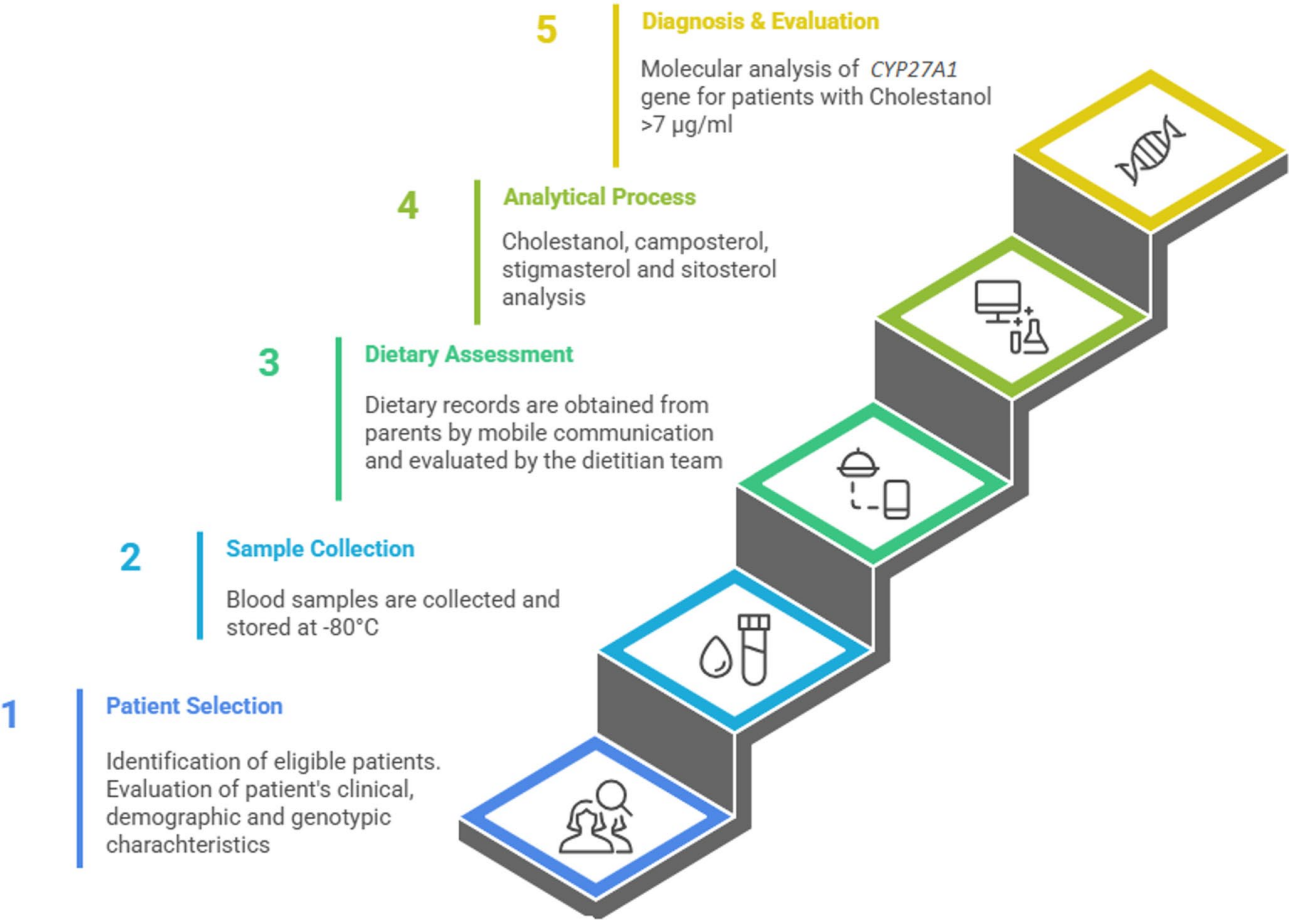
Study workflow and sampling timeline

This study followed a sequential approach starting with the identification of participants who met the inclusion criteria. Eligible participants underwent a comprehensive physical examination and were assessed using the Mignarri Suspicion Index, a clinical scoring system designed to identify patients at risk for CTX based on specific clinical and neuroradiological findings (Mignarri et al. 2014). Clinical and demographic data were obtained from patient records. Blood samples were collected in a fasting state, processed by centrifugation and stored at -80 °C for subsequent analysis of phytosterols such as cholestanol, campesterol, stigmasterol and sitosterol. In patients with cholestanol levels above 7 µg/ml (Salen 1971; Leitersdorf et al. 1994), molecular tests for *CYP27A1* gene variants were performed. In addition, dietary intake data were collected from parents to analyze the possible effects of diet on plasma phytosterol levels. The study schedule is shown in Fig. 1.

### Demographic and clinical data collection and dietary intake assessment

Patient characteristics were systematically recorded to assess demographic data, previous molecular analyses, neuroimaging results, ongoing nutritional and medical treatments, and secondary comorbidities. Each patient then underwent a detailed neurological and systemic examination, followed by calculation of the Mignarri Suspicion Index to assess the likelihood of CTX.

Metabolic dietitians obtained retrospective five-day food consumption records from caregivers via online interviews to assess the ASD patients’ dietary intake. All macro- and micronutrient intakes, including daily energy, carbohydrate, fat and protein intakes, were calculated using the Nutrition Information System (BeBIS^®^) 8.0, and ingredients were calculated based on daily calorie intake (DCI). In addition, consumption of foods high in phytosterols, such as nuts, vegetable oils, cacao, whole grains and avocado, was specifically assessed to determine potential dietary contributions to sterol measurements.

### Procedure

For the biochemical analysis, the patients were informed about the procedure and provided consent for the blood sample to be taken. The blood samples were taken after an overnight fasting period. Samples were immediately processed by centrifugation and stored at − 80 °C under strict cold chain protocols to ensure sample integrity. Cholestanol, campesterol, stigmasterol and sitosterol levels were measured. To exclude secondary sterol elevations related to cholestasis, concurrent routine liver biochemistry (aspartate aminotransferase, alanine aminotransferase, gamma-glutamyl transferase, alkaline phosphatase, total and direct bilirubin) obtained during clinical follow-up at the time of sampling was reviewed, and participants with laboratory evidence of cholestasis were excluded. Plasma samples with visible hemolysis were excluded from analysis.

#### Chemical materials

Chemical materials used in this study included cholestanol, campesterol, stigmasterol and sitosterol standards, as well as hexane, ethanol, potassium hydroxide (KOH), pyridine and N, O-Bis-(Trimethylsilyl) Trifluoroacetamide (BSTFA), all of which were obtained from Sigma-Aldrich and used at analytical grade.

#### Sample collection and preparation

Samples collected in EDTA tubes were centrifuged at 3000 rpm for 5 min to separate the plasma and stored at -20 °C. Prior to analysis, 100 µL of plasma was brought to room temperature and 1 mL of a 4% KOH + 90% ethanol solution was added. The mixture was vortexed and incubated at 60 °C for 15 min. After cooling to room temperature, 1 mL of deionized water and 4 mL of hexane were added for extraction. The organic phase was separated by centrifugation at 4000 rpm for 2 min and evaporated at 30 °C under nitrogen. Subsequently, 50 µL BSTFA and 50 µL pyridine were added, vortexed, and incubated at 60 °C for 30 min. After cooling to room temperature, the samples were transferred to vials for GC/MS analysis.

#### Chromatographic conditions

Analyses were performed using a Hewlett Packard 5973 Mass Selective Detector, an HP6890 GC System, and an Agilent 7683 series injector system and controlled by HP ChemStation software. The separation was performed using an HP-5MS capillary column (30 × 0.25 × 0.25 mm). The temperature of the injector and the transfer line was maintained at 290 °C. Helium was used as the carrier gas. The column temperature was initially set at 150 °C for 2 min, then increased at a rate of 30 °C/min to 270 °C, followed by an increase of 10 °C/min to 290 °C, where it was maintained for 7 min. The sample solutions were then analyzed (Lee et al. 2019).

### Data analysis

Patients with cholestanol levels of more than 7 µg/ml underwent molecular genetic analysis, independent of their Mignarri Suspicion Index. A *CYP27A1* gene analysis was performed and patients with biallelic variants were diagnosed with CTX.

Reference intervals for other phytosterols were defined as < 8 µg/mL for campesterol, < 0.5 µg/mL for stigmasterol and < 15 µg/mL for sitosterol. Individuals with sitosterol concentrations above the reference interval were further evaluated for sitosterolemia by sequencing *ABCG5* and *ABCG8* to exclude secondary etiologies.

#### Statistical analysis

Statistical analyses were performed using Jamovi software (version 2.6.44; The Jamovi Project, 2025). Descriptive statistics were expressed as mean ± standard deviation (SD) or median (25th – 75th percentiles), depending on the distribution of the data. Categorical variables were presented as frequencies and percentages. Normality of continuous variables was tested using the Shapiro–Wilk test. For comparisons between groups, independent-samples t-tests were used for normally distributed variables, while the Mann–Whitney U-test was applied for non-normally distributed data. Associations between continuous variables were analyzed using the Pearson’s correlation coefficient, and relationships between categorical variables were assessed using the chi-square (χ²) test. A two-tailed p-value ≤ 0.05 was considered statistically significant.

## Results

A total of 173 participants were enrolled in the study, including 103 children diagnosed with ASD and 70 age-matched typically developed children. The median age in the ASD group was 8.08 years (IQR 5.74–10.40), while in the control group it was 7.79 years (IQR 5.18–11.80), with no statistically significant difference between the groups (*p* = 0.810). The ASD cohort included 91 males (88.3%) and 12 females (11.7%) while the control group included 45 males (64.3%) and 25 females (35.7%) (*p* < 0.001). Elevated plasma cholestanol concentrations (≥ 7 µg/mL) were detected in 27 children with ASD (26.2%), while no elevations were observed in controls. Also, in the same population the Mignarri Suspicion Index was ≥ 100 in 2/27 and 50 in 25/27, largely driven by psychiatric manifestations (Supplementary Table S1). *CYP27A1* gene sequencing was performed in all patients with elevated cholestanol levels. One patient was found to have a heterozygous variant of uncertain significance (VUS), but no biallelic pathogenic variants were identified. One patient had sitosterol level above the reference threshold (> 15 µg/mL), but targeted sequencing did not identify biallelic variants in *ABCG5* or *ABCG8*. No participant was receiving lipid-lowering therapy at the time of sterol measurement.

### Evaluation and comparison of plasma sterol levels between the patient and control groups

**Fig. 2:**
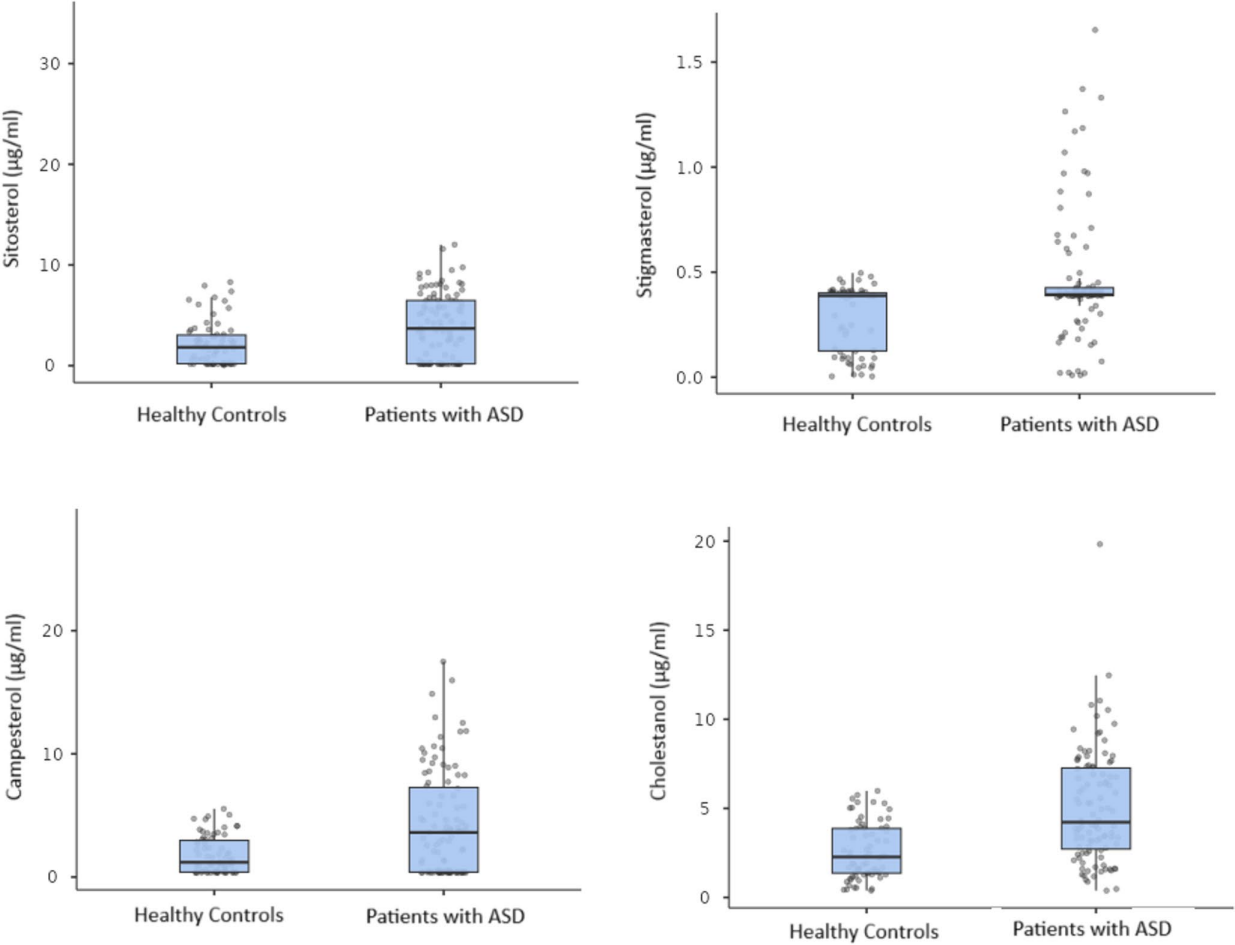
Plasma sterol concentrations in patients with ASD and healthy controls. Box-and-jitter plots display the median and interquartile range (IQR); whiskers indicate 1.5×IQR; points represent individual participants (µg/ml). Median levels were higher in ASD for all sterols: cholestanol, campesterol, sitosterol and stigmasterol. p-values from two-sample tests, as detailed in Methods, were as follows: cholestanol *p* < 0.001, campesterol *p* = 0.001, sitosterol *p* < 0.001, stigmasterol *p* = 0.001. *(Abbreviations: ASD*,* autism spectrum disorder; HC*,* healthy controls)*

The analysis of plasma sterols revealed significantly higher median concentrations of cholestanol, campesterol, sitosterol and stigmasterol in the ASD group compared to the typically developed children. The differences between the groups were statistically significant for all sterols: cholestanol (*p* < 0.001), campesterol (*p* = 0.001), sitosterol (*p* < 0.001) and stigmasterol (*p* = 0.001). The statistical data are shown in Table 1; Fig. 2.

**Table 1:** Comparison of plasma sterol levels between patients with ASD and healthy controls

Sterol (µg/ml)	Patients with ASD	Healthy controls	*p*-value	Mean difference (95% CI)*
Cholestanol	4.22 (2.72–7.27)	2.27 (1.36–3.87)	**< 0.001**	−2.10 (− 2.83 to − 1.39)
Campesterol	3.62 (0.40–7.27)	1.20 (0.40–2.98)	**0.001**	−1.42 (− 2.67 to − 0.06)
Sitosterol	3.69 (0.18–6.47)	1.82 (0.19–3.04)	**< 0.001**	−1.67 (− 2.73 to − 0.28)
Stigmasterol	0.393 (0.388–0.426)	0.388 (0.125–0.401)	**0.001**	−0.038 (− 0.169 to − 0.005)

Mann–Whitney *U* test

* Mean (ASD- control) with bias-corrected 95 % confidence interval

### Dietary energy and fat intake in relation to cholestanol status

Dietary intake data were available for 75 of 103 children in the ASD cohort, including all 24 participants with plasma cholestanol levels above the diagnostic cut-off (≥ 7 µg/mL) and 51 participants with levels below the cut-off. A comparative analysis between these two subgroups revealed no statistically significant differences in total daily energy intake or macronutrient composition, including carbohydrate, protein and fat content (*p* > 0.05). The frequency of consumption of seven predefined food categories high in phytosterols - nuts, vegetable oils, whole grains, legumes, cacao products, seeds and avocado - also did not differ significantly between children with elevated and normal cholestanol level (*p* > 0.05). The data for statistical comparison of dietary energy, macronutrient intake and consumption of foods high in phytosterols between ASD patients with elevated and normal cholestanol levels are shown in Table 2.

**Table 2:** Comparison of dietary energy, macronutrient intake and consumption of foods high in phytosterols in patients with ASD with elevated and normal cholestanol levels

	Patients with ASD with cholestanol < 7 µg/ml (*n* = 51*)	Patients with ASD with cholestanol ≥ 7 µg/ml (*n* = 24)	*p* value
	mean ± SD or *n* (%)		
Energy (kcal/day)	1495 ± 357	1470 ± 309	0.765^a^
Dietary Fat Intake (% DCI)	43.0 ± 11.2	44.8 ± 8.2	0.487^a^
*Categorical variables (proportion “yes”)*			
Regular nut consumption	44/51 (86.3%)	22/24 (91.7%)	0.616^b^
Use of vegetable oils	50/51 (98.0%)	24/24 (100%)	0.272 ^b^
Cocoa intake	35/51 (68.6%)	16/24 (66.7%)	0.865 ^b^
Shellfish consumption	2/51 (3.9%)	1/24 (4.2%)	0.216 ^b^
Whole-grain products	40/51 (78.4%)	19/24 (79.2%)	0.942 ^b^
Seed consumption	27/51 (52.9%)	11/24 (45.8%)	0.566 ^b^
Avocado consumption§	10/50 (20.0%)	8/24 (33.3%)	0.211 ^b^

*ASD* autism spectrum disorder, *SD* standard deviation, *DCI *daily caloric intake

^a^Student’s t-test, ^b^Pearson’s χ² test

### Assessment of factors associated with plasma sterol levels in patients with ASD

Within the ASD cohort, plasma cholestanol concentrations and the frequency of elevated cholestanol (≥ 7 µg/mL) did not differ by sex (*p* > 0.05), and no correlation was observed between sex and plasma cholestanol concentration (*r* = -0.105, *p* = 0.171).

**Fig. 3:**
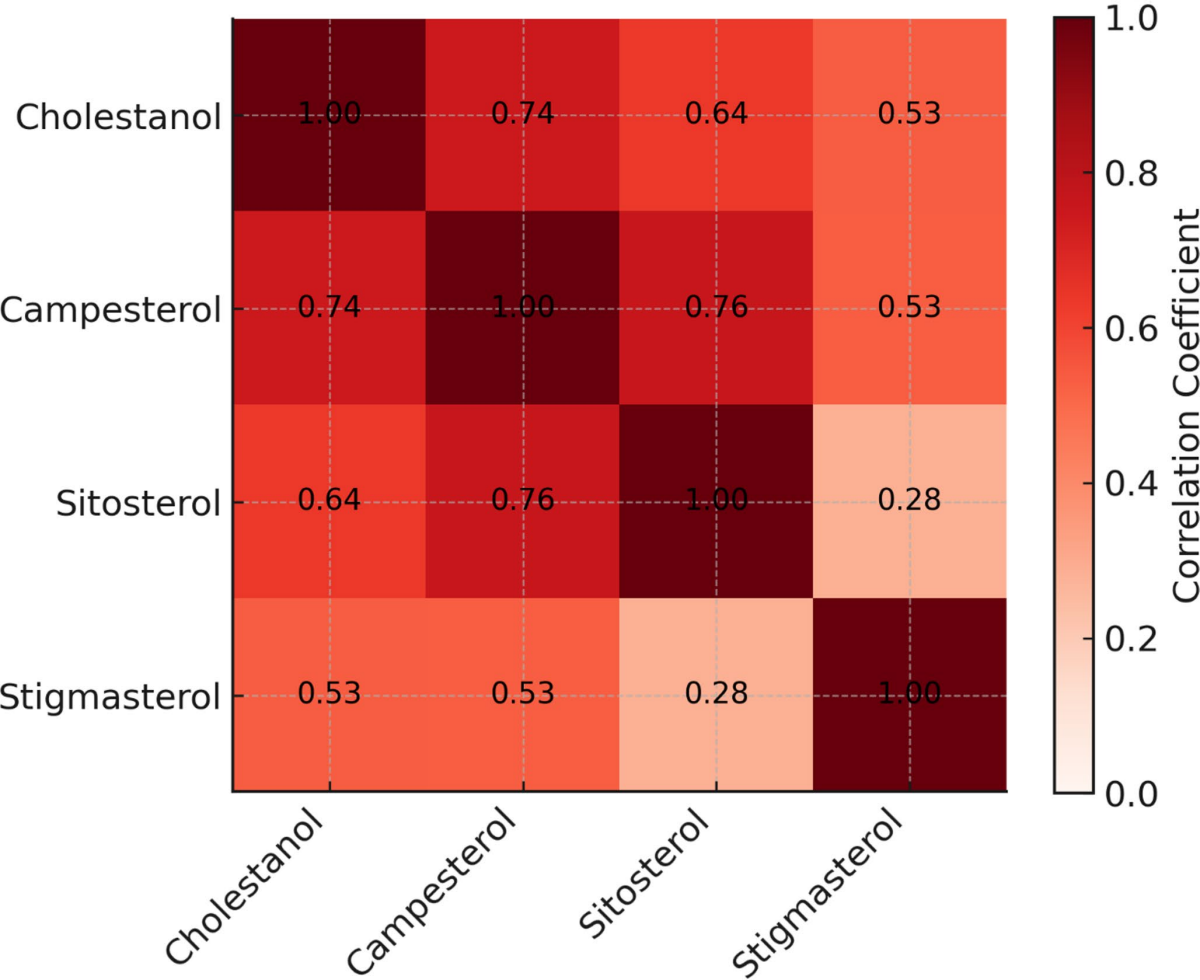
Heatmap visualization of correlation analysis between plasma sterol levels. Each cell shows the Pearson correlation coefficient (r); the legend indicates coefficient magnitude (0–1). Strong positive correlations were observed for cholestanol-campesterol (*r* = 0.744, *p* < 0.001), cholestanol-sitosterol (*r* = 0.635, *p* < 0.001) and campesterol-sitosterol (*r* = 0.761, *p* < 0.001); moderate correlations for cholestanol-stigmasterol (*r* = 0.533, *p* < 0.001) and campesterol-stigmasterol (*r* = 0.528, *p* < 0.001); a weaker association for sitosterol-stigmasterol (*r* = 0.283, *p* = 0.015). Exact values are provided in Table 3

The correlation analysis among the patients with ASD revealed a strong correlation between the measured sterol fractions. Cholestanol levels showed strong positive correlations with campesterol (*r* = 0.744, *p* < 0.001) and sitosterol (*r* = 0.635, *p* < 0.001) and a moderate correlation with stigmasterol (*r* = 0.533, *p* < 0.001). Similarly, campesterol was strongly correlated with sitosterol (*r* = 0.761, *p* < 0.001) and moderately correlated with stigmasterol (*r* = 0.528, *p* < 0.001). A weaker but statistically significant correlation was observed between sitosterol and stigmasterol (*r* = 0.283, *p* = 0.015). The correlation analysis is summarized in Table 3 and a heatmap visualisation of the relevant data is also shown in Fig. 3.

In contrast, correlation analyses revealed no significant associations between plasma sterol concentrations and total daily energy intake or any of the seven predefined high-phytosterol food group variables (all *p* > 0.05).

**Table 3:** Correlation analysis between plasma sterol levels and dietary intake

Cholestanol	Cholestanol	Campesterol	Stigmasterol	Sitosterol
	1	0.744^***^	0.533^***^	0.635^***^
**Campesterol**		1	0.528^***^	0.761^***^
**Stigmasterol**			1	0.283^***^
**Sitosterol**				1

The correlation coefficients are shown

*** *p* < 0.001

## Discussion

In this study, we performed a cross-sectional plasma cholestanol screening in 103 children with ASD to investigate the potential cases of undiagnosed CTX and explore associated sterol metabolism patterns. Although 27 ASD patients (26.2%) had elevated cholestanol levels (≥ 7 µg/mL), no cases of CTX were confirmed by *CYP27A1* gene analysis. However, an unexpected observation was the concurrent elevation of cholestanol and additional sterol fractions - particularly campesterol, sitosterol and stigmasterol - in these patients, with levels significantly higher than in typically developed children. Importantly, the dietary assessments revealed no significant differences in total fat intake or consumption of foods high in phytosterols between ASD patients with elevated and normal cholestanol levels, suggesting that the elevated sterol levels observed are unlikely to be due to dietary or environmental factors. These results support the hypothesis that subtle alterations in bile acid metabolism may be present in a subset of children with ASD. Our findings suggest that cholestanol-based CTX screening in clinically unselected ASD cohorts may have a low diagnostic yield; targeted evaluation guided by additional suggestive clinical features may be more informative. However, our results indicate that bile acid-related metabolic pathways may be future targets for ASD research and therapeutic exploration.

The clinical course of CTX is heterogeneous and the presentation varies according to age: chronic diarrhea and persistent neonatal cholestasis often occur in infancy, intellectual disability and juvenile cataract in early childhood, while progressive neurologic findings - cerebellar, pyramidal and extrapyramidal signs - often do not appear until adolescence or adulthood (Federico and Gallus 1993; Mignarri et al. 2014). Although earlier studies considered neurological deterioration predominantly in adulthood, more recent studies have emphasized that neurological symptoms can also occur in childhood (Wong et al. 2018; Zubarioglu et al. 2024). Early diagnosis is critical, as timely administration of CDCA can prevent or stabilize neurological impairment (Stelten et al. 2019, 2021; Verrips et al. 2020). However, due to significant clinical overlap with other neurodegenerative diseases, CTX is often underdiagnosed or misdiagnosed, resulting in an average diagnostic delay of nearly two decades (Mignarri et al. 2014; Zubarioglu et al. 2024). In response, targeted screening strategies have been proposed in at-risk populations, particularly patients with juvenile cataracts, where CTX diagnosis has actually been made in a subset of cases with a prevalence between 0.99 and 3.3% (Freedman et al. 2019, 2023; Atilla et al. 2021; Fernández-Eulate et al. 2022). However, large-scale screening measures for CTX are largely limited to cataract populations.

Previous case reports and observational studies have described the co-occurrence of CTX and ASD, suggesting that ASD may represent an early neurodevelopmental manifestation of CTX in rare cases (Stelten et al. 2018; Zubarioglu et al. 2024). Behavioral and psychiatric symptoms, including autism-like features, may not only be part of the broader CTX phenotype, but may also respond positively to CDCA replacement therapy if initiated early, indicating a potentially reversible component of neuropsychiatric involvement (Stelten et al. 2018). However, ASD populations have not been routinely included in CTX screening strategies and few literatures have examined this association, despite the possibility that subtle metabolic disturbances may contribute to the ASD phenotype. The limited number of publications addressing CTX screening in individuals with ASD emphasizes the importance of further studies in this under-researched area (Karadag et al. 2024).

To date, the only study in the literature to screen for CTX in an ASD population is a prospective observational study that included 101 pediatric patients diagnosed with ASD who also had at least one systemic or psychiatric symptom suggestive of CTX according to the Mignarri Suspicion Index (Karadag et al. 2024). All participants underwent direct *CYP27A1* gene sequencing without prior plasma sterol screening, and two patients (1.9%) were found to carry biallelic variants of uncertain significance (VUS). Although plasma cholestanol cut-off values were not assessed or optimized due to the lack of pre-screening, the study highlighted that targeted genetic testing in clinical high-risk ASD subgroups, especially in patients with early systemic and psychiatric comorbidities, may facilitate early identification of CTX and enable timely CDCA treatment to prevent irreversible neurological deterioration.

In our study, a complementary biochemistry-based strategy was used to explore CTX risk in a similarly sized, clinically unselected cohort of 103 children diagnosed with ASD, using measurement of plasma cholestanol measurement as an initial screening step and performing *CYP27A1* gene analysis only in those with elevated levels. Elevated plasma cholestanol levels were detected in 27 participants; however, no case of CTX was confirmed by genetic analysis. The absence of a CTX diagnosis in this cohort may suggest that individuals with isolated ASD may not be an appropriate target group for CTX screening, especially in the absence of characteristic features such as juvenile cataract, chronic diarrhea, or tendon xanthomas. However, our results revealed an unexpected secondary observation regarding plasma sterol profiles within the ASD population. A notable proportion of ASD patients (26%) had plasma cholestanol concentrations above 7 µg/ml and mean plasma cholestanol levels were significantly higher in the ASD group compared to typically developed children (*p* < 0.001). False high cholestanol levels may occur due to high dietary phytosterol intake, contamination of samples or concurrent hepatic conditions such as cholestasis or sitosterolemia. However, in our study, possible causes of secondary elevation were systematically excluded. The effects of high nutritional phytosterol intake were ruled out by dietary intake records and no concomitant hepatic or pharmacologic confounders were identified. This observation raises the question of whether subtle disturbances of bile acid metabolism are more common in ASD than previously thought and suggests the need to investigate the possible role of sterol metabolism abnormalities in the pathophysiology of ASD.

Sterol metabolism provides an informative surrogate marker for bile acid metabolism, as both metabolic pathways start from cholesterol and involve overlapping enzymatic steps (Russell 2003). In liver tissue, cholesterol is first converted to 7-α-hydroxycholesterol by cholesterol 7α-hydroxylase (CYP7A1), which is the rate-limiting step of the classical bile acid synthesis pathway (Federico and Gallus 1993; Nie et al. 2014). Subsequent enzymatic reactions ultimately produce the primary bile acids cholic acid and chenodeoxycholic acid. Disruptions at any point in this cascade - whether due to enzyme deficiencies, mitochondrial dysfunction or regulatory impairments - can lead to an accumulation of metabolic intermediates and a diversion of cholesterol into alternative pathways (Nie et al. 2014). This diversion leads to increased concentrations of neutral sterols such as cholestanol and phytosterols such as campesterol and stigmasterol (de Sain-van der Velden et al. 2008). Elevated serum sterol concentrations may therefore serve as an accessible biochemical indicator of impaired bile acid synthesis contextualizing our findings within a broader framework of altered sterol and bile acid homeostasis in individuals with ASD.

Bile acid biosynthesis is a tightly regulated, multi-step metabolic pathway involving numerous hepatic enzymes, and even mild impairments of this cascade, whether through genetic polymorphisms, impaired transcriptional control or alterations in the gut microbiota, can disrupt sterol homeostasis (Yang et al. 2024). Untargeted urine metabolomics in acute intermittent porphyria have revealed a disturbance of bile acid metabolism, which may contribute to the prediction of clinical attacks (Lefebvre et al. 2025). Similarly, in Niemann-Pick disease type C, specific glycine-conjugated bile acid species have been found to be significantly elevated and now serve as sensitive diagnostic biomarkers (Jiang et al. 2016).

Beyond CTX, several disorders of cholesterol biosynthesis can present with neurodevelopmental phenotypes and may also disrupt bile acid homeostasis. Smith-Lemli-Opitz syndrome (SLOS), which results from impaired cholesterol biosynthesis, is well recognized as being associated with autistic features (Sikora et al. 2006). Abnormal bile acid synthesis has been reported in SLOS, and bile acid-based interventions have been explored in more severely affected individuals (Svoboda et al. 2012). Recent studies have also highlighted a potential link between bile acid metabolism and ASD. In a population-based case-control study, maternal mid-pregnancy serum of women whose children were later diagnosed with ASD was shown to display elevated levels of metabolites involved in bile acid biosynthesis, suggesting that alterations in maternal bile acid profiles may play a role in early fetal neurodevelopment (Ritz et al. 2020). This is supported by an experimental study using a mouse model of ASD that found evidence of impaired bile acid production in the liver, again suggesting a possible link between bile acid metabolism and neurodevelopment (Cao et al. 2023). Consistent with these findings, a large Swedish population-based study found an increased risk of ASD in children born to mothers with intrahepatic cholestasis during pregnancy, providing further evidence of elevated maternal bile acid levels as a potential factor in susceptibility to neurodevelopment (Chen et al. 2024). Overall, these findings suggest that intrinsic disturbances in bile acid metabolism, whether maternal, hepatic or systemic in origin, represent a contributing mechanism in ASD and warrant further investigation as potential biomarkers or therapeutic targets.

This possible association between ASD and bile acid metabolism raises the question of where it comes from. This could be caused or exacerbated by abnormalities in the gut microbiome, a common feature observed in affected individuals (Pulikkan et al. 2019). Intestinal bacteria play a central role in the deconjugation, transformation and enterohepatic circulation of bile acids. Dysbiosis may lead to altered bile acid pools, resulting in impaired reabsorption and accumulation of intermediate sterols (Jones et al. 2014). This is particularly important as infantile diarrhea, as seen in CTX, indicates malabsorption of bile acids; similarly, a subset of idiopathic ASD cases also have chronic diarrhea, which could indicate subtle bile acid diarrhea or malabsorption. In addition, gut-derived metabolites may modulate systemic inflammation and metabolic signaling, further influencing brain development. Aberrant bile acid profiles may also result in failure to activate gut–brain signaling pathways, such as those involving FXR and TGR5, which normally support neurodevelopment (Pols et al. 2011). In children with ASD, gastrointestinal symptoms often co-occur with behavioral symptoms, and altered microbial profiles have been associated with changes in lipid metabolites (El-Ansary et al. 2020). Therefore, the gut–liver–brain axis may act as both a source and mediator of bile acid and sterol imbalance in ASD, providing a unifying framework that integrates environmental, microbial and host-specific metabolic contributions to neurodevelopmental risk.

In addition to the elevated cholestanol levels, we observed elevated levels of plant sterols in some children with ASD. These phytosterols are provided with food and serve as surrogate biomarkers for intestinal cholesterol absorption. However, prior studies evaluating plant sterols in ASD have reported heterogeneous findings. For example, Tierney et al. quantified sitosterol and cholesterol precursors in an ASD cohort and reported that sitosterol concentrations were generally within laboratory reference ranges, with only mild elevations in a small subset (Tierney et al. 2021). In our cohort, sitosterol likewise remained within the reference interval at the group level (median 3.69 µg/mL), yet it was modestly higher than in typically developed controls, indicating a subtle shift rather than overt sitosterolemia; only one child had sitosterol > 15 µg/mL and *ABCG5/ABCG8* sequencing was negative. In particular, elevated plasma levels of plant sterols have been reported in untreated CTX patients and shown to decrease after bile acid replacement therapy with CDCA (Mignarri et al. 2016). In this study, the authors hypothesized that high cholic acid levels prior to treatment may improve sterol absorption, a mechanism that may be reversible with CDCA. However, none of the participants in our cohort were diagnosed with CTX; nevertheless, similar elevations in phytosterols were noted. This finding raises the question of whether some individuals with ASD have unique alterations in sterol absorption or sterol metabolism that may be related to bile acid composition, intestinal transporter activity, or gut microbiota. Although dietary intake may influence sterol levels, our analysis considered dietary sterol sources and excluded secondary dietary factors. These results suggest that phytosterol profiling may provide additional insight into cholesterol homeostasis and metabolic heterogeneity within populations with ASD.

This study has several limitations. First, although our sample size of 103 children with ASD is relatively large compared to many previous studies, it remains small for a screening study aimed at assessing the prevalence of a rare disorder such as CTX. Second, although we determined plasma sterol levels as a proxy for bile acid metabolism, we did not evaluate the full spectrum of bile acid intermediates and elements of the metabolic pathway, which would have allowed a more comprehensive assessment of subtle disorders of bile acid synthesis. Third, dietary recalls were collected only in ASD participants; therefore, dietary energy/macronutrient intake and phytosterol-rich food consumption could not be directly compared between ASD and control children. Lastly, although significant changes in sterol levels were found, we did not investigate potential associations between these biochemical findings and autism symptom severity using standardized clinical rating scales such as the Childhood Autism Rating Scale (CARS).

## Conclusion

Our results suggest that CTX screening based solely on an ASD diagnosis may have limited yield; prioritizing individuals with additional suggestive clinical features may improve the efficiency of case detection. Disruptions in the sterol and bile acid pathways may contribute to the underlying metabolic basis of ASD, warranting further investigation. 

## Supplementary Information

Below is the link to the electronic supplementary material.

Supplementary Material 1 (DOCX. 35.6 KB)

## Data Availability

The data that support the findings of this report are available from the corresponding author upon reasonable request.

## Declarations

### Use of AI and AI-assisted Technologies Statement

ChatGPT was used as an English proofreading service.
