# Supplementary material for: Plasma sterol profiling in autism spectrum disorder: insights from cerebrotendinous xanthomatosis screening and beyond
Source: Metab Brain Dis. 2026 Mar 18;41(1):60. doi: 10.1007/s11011-026-01827-7 (PMC12999745; doi:10.1007/s11011-026-01827-7)
Supplement: Supplementary file 1 — Supplementary Material 1 (DOCX. 35.6 KB) [file 11011_2026_1827_MOESM1_ESM.docx]

**Supplementary Table S1: Mignarri Suspicion Index scores for ASD patients with elevated cholestanol levels**

| Mignarri Suspicion Index Item (score) | P1 | P2 | P3 | P4 | P5 | P6 | P7 | P8 | P9 | P10 | P11 | P12 | P13 | P14 |
| --- | --- | --- | --- | --- | --- | --- | --- | --- | --- | --- | --- | --- | --- | --- |
| Sibling with CTX (100) | − | − | − | − | − | − | − | − | − | − | − | − | − | − |
| Tendon xanthomas (100) | − | − | − | − | − | − | − | − | − | − | − | − | − | − |
| Consanguineous parents (50) | + | − | − | − | − | − | − | − | − | − | − | − | − | − |
| Juvenile cataract (50) | − | − | − | − | − | − | − | − | − | − | − | − | − | − |
| Childhood-onset chronic diarrhea (50) | − | − | − | − | − | − | − | − | − | − | − | − | − | − |
| Prolonged unexplained neonatal jaundice (50) | − | + | − | − | − | − | − | − | − | − | − | − | − | − |
| Cerebellar ataxia and/or spastic paraparesis (50) | − | − | − | − | − | − | − | − | − | − | − | − | − | − |
| Dentate nucleus signal change in neuroimaging (50) | − | − | − | − | − | − | − | − | − | − | − | − | − | − |
| Intellectual disability and/or neuropsychiatric involvement (50) | + | + | + | + | + | + | + | + | + | + | + | + | + | + |
| Early osteoporosis (25) | − | − | − | − | − | − | − | − | − | − | − | − | − | − |
| Epilepsy (25) | − | − | − | − | − | − | − | − | − | − | − | − | − | − |
| Parkinsonism (25) | − | − | − | − | − | − | − | − | − | − | − | − | − | − |
| Polyneuropathy (25) | − | − | − | − | − | − | − | − | − | − | − | − | − | − |
| Total score | **100** | **100** | **50** | **50** | **50** | **50** | **50** | **50** | **50** | **50** | **50** | **50** | **50** | **50** |

| Mignarri index item (score) | P15 | P16 | P17 | P18 | P19 | P20 | P21 | P22 | P23 | P24 | P25 | P26 | P27 |
| --- | --- | --- | --- | --- | --- | --- | --- | --- | --- | --- | --- | --- | --- |
| Sibling with CTX (100) | − | − | − | − | − | − | − | − | − | − | − | − | − |
| Tendon xanthomas (100) | − | − | − | − | − | − | − | − | − | − | − | − | − |
| Consanguineous parents (50) | − | − | − | − | − | − | − | − | − | − | − | − | − |
| Juvenile cataract (50) | − | − | − | − | − | − | − | − | − | − | − | − | − |
| Childhood-onset chronic diarrhea (50) | − | − | − | − | − | − | − | − | − | − | − | − | − |
| Prolonged unexplained neonatal jaundice (50) | − | − | − | − | − | − | − | − | − | − | − | − | − |
| Cerebellar ataxia and/or spastic paraparesis (50) | − | − | − | − | − | − | − | − | − | − | − | − | − |
| Dentate nucleus signal change in neuroimaging (50) | − | − | − | − | − | − | − | − | − | − | − | − | − |
| Intellectual disability and/or neuropsychiatric involvement (50) | + | + | + | + | + | + | + | + | + | + | + | + | + |
| Early osteoporosis (25) | − | − | − | − | − | − | − | − | − | − | − | − | − |
| Epilepsy (25) | − | − | − | − | − | − | − | − | − | − | − | − | − |
| Parkinsonism (25) | − | − | − | − | − | − | − | − | − | − | − | − | − |
| Polyneuropathy (25) | − | − | − | − | − | − | − | − | − | − | − | − | − |
| Total score | **50** | **50** | **50** | **50** | **50** | **50** | **50** | **50** | **50** | **50** | **50** | **50** | **50** |

*Intellectual disability and/or neuropsychiatric involvement (50) was marked present for all patients because ASD was the inclusion diagnosis. Two patients reached a total score of 100 due to one additional item (one with prolonged unexplained neonatal jaundice and one with parental consanguinity).*
